# Y-configuration double-stent-retriever thrombectomy for refractory thrombus in middle cerebral artery bifurcation: A case report

**DOI:** 10.1097/MD.0000000000024993

**Published:** 2021-03-19

**Authors:** Changchun Jiang, Yuechun Li, Fei Hao, Junfeng Yang, Baojun Wang, Yu Fan

**Affiliations:** Department of Neurology, Baotou Central Hospital, Baotou, Inner Mongolia, China.

**Keywords:** acute ischemic stroke, double stent-retrievers, middle cerebral artery bifurcation, thrombectomy

## Abstract

**Rationale:**

Stent retriever mechanical thrombectomy is a recommended treatment for acute ischemic stroke. However, refractory thrombus in artery bifurcation can reduce the rate of successful revascularization.

**Patient concerns:**

A 72-year-old male, owing to the acute onset of almost complete right-sided hemiplegia and global aphasia, received bridging therapy. National Institutes of Health Stroke Scale score was 16 at the time of admission.

**Diagnoses:**

Cerebral digital subtraction angiography revealed occlusion of the M1 segment of the left MCA.

**Interventions:**

Thrombectomy with 3 passes of the Solitaire FR device (Medtronic, Minneapolis, MN) was unsuccessful. Two stent retrievers were inserted in parallel by one microcatheter access point to each M2 branch, and then both stents were gradually retrieved out of the catheter while continuous suction was maintained.

**Outcomes:**

After thrombectomy, subsequent follow-up angiograms showed mTICI 3 reperfusion of MCA. The patient has mRS 2 at discharge and the 3-month mRS score after stroke is 1 score.

**Lessons:**

The presented Y-configuration double-stent-retriever thrombectomy technique constitutes a safe and effective rescue treatment method for refractory thrombus in MCA bifurcation.

## 1 Introduction

Stent retriever mechanical thrombectomy is a safe and effective treatment for acute ischemic stroke patients with large-vessel occlusion. Recently published randomized trials and real-world registry studies have shown that the rate of successful revascularization, defined as a mTICI score of 2b/3, has increased from 65.7% to 88%.^[1–8]^ Therefore, approximately one-third of patients with acute occlusion of large vessels cannot achieve revascularization by way of mechanical thrombectomy. One of the reasons for failure of revascularization is refractory thrombus in artery bifurcation. Here, we describe the use of double Solitaire FR stent retrievers (Medtronic, Minneapolis, MN) for mechanical thrombectomy of MCA bifurcation after failed thrombectomy using a single stent retriever. The Ethics Committee approved the case report, which was in compliance with the Helsinki Declaration.

## 2 Case report

A 72-year-old male with a history of hypertension and ischemic stroke arrived at our hospital's comprehensive stroke center within 2 hours owing to the acute onset of almost complete right-sided hemiplegia and global aphasia. Neurological examination on admission showed few recognizable words and no spoken language, total gaze paresis, and complete paralysis of the right side of the body. National Institutes of Health Stroke Scale score was 16 at the time of admission. Noncontrast computed tomography of the brain did not reveal an observation of acute cerebral infarction, early signs of infarction, or hemorrhage. At 134 minutes after stroke onset, the patient received thrombolytic therapy with intravenous recombinant tissue plasminogen activator at a dose of 0.9 mg/kg according to his weight, with 10% given as a bolus and rest administered over 60 minutes. The patient did not improve by the end of recombinant tissue plasminogen activator infusion. Noncontrast computed tomography before bridging therapy revealed that his Alberta Stroke Program Early CT Score value was 7. Cerebral digital subtraction angiography with conscious sedation revealed left proximal common carotid artery severe tortuous and occlusion of the M1 segment of the left MCA. The anterior and left posterior communicating arteries did not compensate for the blood supply area of the left middle cerebral artery effectively. The American Society of Interventional and Therapeutic Neuroradiology and Society of Interventional Radiology grade was 3. The informed consent was signed by the legal representatives prior to endovascular therapy.

**Figure 1:**
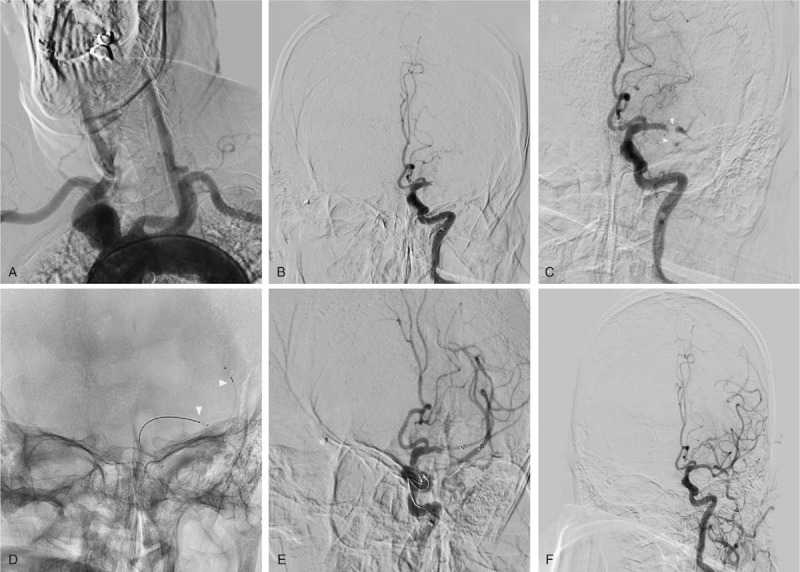
(A) Aortic arch angiography showing that the left proximal common carotid artery was severely tortuous. (B) A frontal angiogram showing an occluded left MCA. (C) Thrombectomy with 3 passes of the Solitaire FR was unsuccessful; arrowheads show the left MCA bifurcation clot. (D) Anteroposterior image showing the Solitaire FR 4 × 15 stent retriever (arrow) in the superior MCA trunk; the proximal end of the stent did not cover the bifurcation. (E) Anteroposterior image showing the kissing-Y stent technique. (F) Frontal angiogram showing complete recanalization of the left MCA.

Endovascular treatment was as follows. A Navien 072 intracranial support catheter (ev3, Plymouth, MN) was introduced into the cavernous segment of the left internal carotid artery. Then, a Rebar 18 microcatheter (ev3, Plymouth, MN) was navigated over the Traxcess-14 (MicroVention, Aliso Viejo, CA), beyond the distal end of the occlusive clot. Thrombectomy with 3 passes of the Solitaire FR device 6 × 30-mm in the inferior MCA trunk was unsuccessful and a left MCA bifurcation clot was observed. At this point, we decided to employ a novel strategy for mechanical thrombectomy incorporating 2 Solitaire FR devices. A Navien 072 catheter could not be placed at the same time at 2 Rebar-18 microcatheters. Therefore, the Rebar-18 microcatheter was first placed into M2 segment of the left MCA through the superior MCA trunk, while a Solitaire FR 4 × 15-mm was placed such that the proximal end of the stent did not cover the bifurcation. Subsequently, the microcatheter was removed completely from the Navien, leaving a bare Solitaire FR inside the Navien. Then, the inferior MCA trunk was catheterized with the same microcatheter and the Solitaire FR 6 × 30-mm was unfolded by withdrawal of the microcatheter. When the microcatheter tip was nearly aligned with the bifurcation, we pulled the first Solitaire FR to engage the clot and continued to withdraw the microcatheter to position the second Solitaire FR in parallel. Then, both stents were slowly pulled together into the Navien under continuous aspiration. As resistance was felt while retracting the stent retriever, the entire assembly was slowly withdrawn under continuous aspiration. Subsequent follow-up angiograms showed mTICI 3 reperfusion of MCA. The interval between groin puncture to final revascularization was 68 minutes. No intracranial hemorrhage or hyperperfusion was found on CT 24 hours after endovascular therapy. The patient has mRS 2 at discharge and the 3-month mRS score after stroke is 1 score (Fig. 1).

## 3 Discussion

Acute ischemic stroke patients with large-vessel occlusion involving bifurcation are difficult to promote sufficient revascularization in using standard mechanical thrombectomy. As such, the use of double stent retrievers for mechanical thrombectomy in these individuals may represent an effective rescue therapy. Large-vessel occlusion involving bifurcation usually has a recalcitrant and high clot burden, leading to a reduction in the possibility of successful recanalization. Typically in these cases, the application of a single stent retriever or other rescue therapies such as intra-arterial thrombolysis, thromboaspiration, balloon angioplasty, or intracranial stent placement may not achieve recanalization of all branches.^[9,10]^ However, in the current case, a single stent retriever could not trap the clot within its stent struts after 3 retrieval attempts. Previous studies^[11–13]^ had reported that the use of double stent retrievers in a Y-configuration can be used for refractory MCA bifurcation. This technique usually requires an 8-French guiding catheter for the internal carotid artery; during the procedure, 2 stent retrievers are inserted in parallel by 2 microcatheter access points to each M2 branch and then both stents are gradually retrieved out of the guiding catheter while continuous suction is maintained. The Y-stent technique includes the crossing-Y and kissing-Y techniques. The latter stent technique is as mentioned above. Conversely, the crossing-Y stent technique requires a wire be inserted through the first stent interstices and into the contralateral branch vessel, increasing the risk of stent entanglement and vessel injury by raising the vessel endothelium during retrieval. The subsequent endovascular treatment plan of this case was kissing-Y-stent-retriever thrombectomy in conjunction with continuous aspiration via Navien 072. Because the left common carotid artery was particularly tortuous, the replacement of an 8-French guiding catheter should be considered difficult. Here, 2 Solitaire FR stents in a kissing-Y formation were unfolded by Navien 072 using 1 microcatheter and the MCA was successfully recanalized.

As a novel mechanical thrombectomy technique, double stent thrombectomy has a high recanalization rate in selected cases and it has many advantages. First, it acts as a temporary bypass by permitting immediate restoration of flow through the clot by expanding the stent within the clot.^[14]^ Second, the double stent led to an increase in the degree of stent expansion, which may reflect the ability of the double stent thrombectomy technique to facilitate the device-clot interaction.^[15]^ Third, double stent thrombectomy technique results in longer device surface, which can enhance the device purchase distal to the clot, increasing the chances of dragging the clot out.^[16]^

The double stent thrombectomy technique has some potential disadvantages, such as vessel injury, dissection, endothelial wall damage, and small artery avulsion, which is potentially higher than that of the simple stent technique.^[15]^ Also, the double stents lead to a significant increase in hospital costs to an already expensive acute stroke treatment. Further studies are required to determine the cost-effectiveness of double stent thrombectomy technique.

In conclusion, after the unsuccessful performance of standard retrieval attempts with a single stent, the Y-configuration double-stent-retriever thrombectomy technique was used and may constitute a rescue treatment for refractory thrombus in MCA bifurcation. This technique could also be applied in refractory carotid terminus or basilar tip occlusions.

## Acknowledgments

We greatly appreciate the participating relevant cliniciansm, the imaging, and laboratory technicians.

## Author contributions

**Conceptualization:** Baojun Wang.

**Data curation:** Yuechun Li, Yu Fan.

**Funding acquisition:** Yu Fan.

**Investigation:** Junfeng Yang.

**Methodology:** Fei Hao, Junfeng Yang.

**Writing – original draft:** Changchun Jiang.

**Writing – review & editing:** Yu Fan.
